# Use of the Neuroform Atlas stent for wide-necked cerebral aneurysms

**DOI:** 10.1038/s41598-023-40725-1

**Published:** 2023-08-22

**Authors:** Hui-Li Gao, Qiu-Ji Shao, Kai-Tao Chang, Li Li, Tian-Xiao Li, Bulang Gao

**Affiliations:** Stroke Center, Henan Provincial People’s Hospital, Zhengzhou University, 7 Weiwu Road, Zhengzhou, Henan China

**Keywords:** Diseases, Health care, Medical research, Neurology

## Abstract

To investigate the effect and safety of the Neuroform Atlas (NFA) stent in stent-assisted coiling of wide-necked intracranial aneurysms, patients with wide-necked intracranial aneurysms were retrospectively enrolled and treated with the NFA stent-assisted coiling. The modified Rankin scale (mRS) grades and Raymond grades were used to assess the clinical outcomes and aneurysm occlusion degrees, respectively, after embolization and at follow-up. Totally, 122 patients were enrolled with 129 wide-necked aneurysms, and forty-nine (40.2%) patients experienced subarachnoid hemorrhage. A total of 134 NFA stents were deployed in all patients. Immediately after endovascular embolization, the Raymond grade was I in 112 (86.8%), II in 8 (6.2%), and III in 9 (7.0%). Complications occurred in 7 (5.7%) patients, including stent displacement in 2 (1.6%) patients, thrombosis and cerebral infarction in 4 (3.3%), and death in 1 (0.8%). Clinical follow-up was performed in 113 (92.6%) patients 6–30 (mean 21) months after embolization, with the mRS grade 0 in 99 (87.6%) patients, 1 in 7 (6.2%), 2 in 5 (4.4%), and 3 in 2 (1.8%). Good prognosis (mRS ≤ 2) was achieved in 111 (98.2%) patients while poor prognosis (mRS > 2) in two (1.8%). Digital subtraction angiography was conducted in 98 (80.3%) patients with 104 (80.6%) aneurysms 6–30 (mean 21) months after embolization. The Raymond grade was grade I in 94 (90.4%) aneurysms, II in 4 (3.8%), and III in 6 (5.8%). Compared with the Raymond grades immediately after embolization, 93 (89.4%) aneurysms disappeared, 9 (8.7%) remained unchanged in the occlusion status, and 2 (1.9%) were recurrent. In conclusion, the NFA stent may have a high aneurysm occlusion rate and a low complication rate in assisting coiling of wide-necked intracranial aneurysms even though further studies are necessary to prove this.

## Introduction

Wide-necked intracranial aneurysms accounted for over 40% of all intracranial aneurysms, and stent-assisted coiling has been widely applied in the treatment of wide-necked intracranial aneurysms, resulting in good effects^1–4^. The Neuroform Atlas (NFA) stent (Stryker Neurovascular, Fremont, CA, USA) is a new generation of the Neuroform stent and is the first low-profile open-cell stent designed to allow for delivery through a microcatheter with a 0.0165 inch inner diameter, better scaffolding because of the smaller cell sizes, improved trackability, and increased conformability to the arterial wall compared with the previous design^5–7^. Initial clinical application of this stent has been conducted and proved its effectiveness^5–7^. Nonetheless, these studies are only of a small case series with a shorter follow-up period. This study was performed in a single large-volume medical center to test the effect and safety of the NFA stent in the treatment of wide-necked intracranial aneurysms in a longer follow-up period.

## Materials and methods

### Subjects

This retrospective one-center study was conducted after approval by the ethics committee of Henan Provincial People’s Hospital, and the informed consent was waived by the same ethics committee because of the retrospective study design. All methods were performed in accordance with the relevant guidelines and regulations. Between August 2020 and February 2023, consecutive patients with wide-necked intracranial aneurysms treated with the NFA stent were retrospectively enrolled. The inclusion criteria were patients with wide-necked (neck diameter > 4 mm or aneurysm dome to neck ratio < 2) intracranial aneurysms confirmed by digital subtraction angiography and treated with the NFA stent-assisted coiling. The exclusion criteria were patients with blood blister-like cerebral aneurysms, dissecting aneurysms, arterial dissection, and combined use of other stents besides the NFA stent.

### Endovascular treatment procedure

All endovascular procedures were conducted under general anesthesia. After puncture of the right femoral artery, a 6F or 8F arterial sheath was inserted and a guiding catheter was navigated to the internal carotid artery (ICA) or vertebral artery. Three-dimensional cerebral angiography was performed for measurement of the aneurysm diameter, neck size, parent artery diameter, and the optimal projection angle for treatment. Then, a micro-catheter was navigated to the aneurysm neck for deployment of an appropriate NFA stent and coiling.

### Periprocedural medication

For unruptured aneurysms, dual antiplatelet therapy was performed with aspirin 100 mg/day and clopidogrel 75 mg/day for 5–7 days before endovascular treatment. After the treatment, dual antiplatelet therapy was continued for 3 months before switching to oral aspirin 100 mg/day alone for 1–2 years. One day before the endovascular treatment, thromboelastography was conducted in all patients to test the body’s reaction to the antiplatelet therapy. Good reaction was achieved when the arachidonic acid was inhibited > 50%, the adenosine diphosphate was inhibited > 30%, and the maximal amplitude of the adenosine diphosphate curve was controlled between 31 and 47 mm. For patients without good reaction in the thromboelastography, small-dose tirofiban was administered intra-procedurally, with 6–8 mg being given intravenously followed by intravenous pumping of 4–6 mg per hour during the procedure and 24–48 h after the procedure. Retest of the thromboelastography was performed before discharge for those who did not have good reaction, and the dosage was adjusted or clopidogrel was replaced by Ticagrelor.

For ruptured aneurysms, the antiplatelet therapy was not administered before endovascular treatment, and tirofiban was used intraprocedurally when deploying a stent, with the initial dosage of 8.0 μg/kg injected intravenously within three minutes followed by tirofiban infusion in the dosage of 0.1 μg/kg/min for maintenance for 24–48 h. Two hours before withdrawal of tirofiban, clopidogrel 300 mg and aspirin 100 mg were administered, and tirofiban was reduced to half the dosage for maintenance. On the second day after withdrawal of tirofiban, dual antiplatelet therapy was conducted for 3–5 days before the test of thromboelastography. After the dual antiplatelet therapy for 3 months, oral use of aspirin 100 mg/day alone was administered for 1–2 years.

### Follow-up and evaluation

Follow-up was performed 3 months after discharge using telephone contact or clinical visit. The modified Rankin Scale (mRS) score was used to assess the clinical prognosis, with good prognosis defined as the mRS ≤ 2 and poor prognosis as mRS > 2. Digital subtraction angiography was conducted for follow-up half a year later to assess the patency of the stent, and the Raymond grades were used to evaluate the occlusion status of the aneurysm, with the Raymond grade I as complete occlusion, grade II as residual neck, and grade III as residual dome^8^. Compared with the occlusion status immediately after embolization, the occlusion status of aneurysms at follow-up was divided into three kinds: disappeared, complete occlusion of the aneurysm; unchanged, no changes in the occlusion status of the aneurysm; recurrence, reopening of the originally completely occluded aneurysms displayed on angiography or the residual neck or dome was enlarged in size.

### Statistical analysis

The statistical analysis was performed with the SPSS 22.0 (IBM, Chicago, IL, USA). Measurement data were presented in mean ± standard deviation and tested with the *t*-test if in the normal distribution or in median and interquartile range and tested with the Mann–Whitney *U* test if not in the normal distribution. Categorical data were expressed as frequency and percentage and tested with the Chi square test. The significance was set as P < 0.05.

## Results

Totally, 122 patients were enrolled, including 49 male and 73 female patients aged 30–75 (56.7 ± 7.1) years (Table 1). Forty-nine (40.2%) patients experienced subarachnoid hemorrhage (SAH), with the Hunt-Hess grade I in five (4.1%) patients, grade II in 34 (69.4%), grade III in six (4.9%), and grade IV in four (3.3%). The other patients were incidentally found. There were 129 cerebral aneurysms in these patients, with a size range 1.2–14.9 (mean 5.3 ± 2.1) mm and a neck size ranging 1.4–8.6 (mean 4.4 ± 2.1) mm. The aneurysms were located in the anterior cerebral artery in 7 (5.4%) patients, anterior communicating artery in 32 (24.8%), ICA ophthalmic segment in 13 (10.1%), posterior communicating artery in 21 (16.3%), middle cerebral artery bifurcation in 31 (24.0%), M2 segment in 4 (3.1%), basilar tip in 16 (12.4%), posterior cerebral artery in 3 (2.3%), and posterior inferior cerebellar artery in 2 (1.6%).

**Table 1:** Demography of the patients.

Variables	Data
M/F	49/73
Age (y)	30–75 (56.7 ± 7.1)
No. of aneurysms (n)	129
Aneurysm size (mm)	1.2–14.9 (5.3 ± 2.1)
< 3 mm	25
3–10 mm	84
> 10 mm	13
Aneurysm neck size (mm)	1.4–8.6 (4.4 ± 2.1)
Aneurysm location (n, %)	129 (100%)
ACA (n, %)	7 (5.4%)
Acom (n, %)	32 (24.8%)
ICA ophthalmic segment (n, %)	13 (10.1%)
ICA Pcom (n, %)	21 (16.3%)
MCA bifurcation (n, %)	31 (24.0%)
MCA M2 (n, %)	4 (3.1%)
Basilar tip (n, %)	16 (12.4%)
PCA (n, %)	3 (2.3%)
PICA (n, %)	2 (1.6%)
SAH (n, %)	49 (40.2%)
HH grade I (n, %)	5 (4.1%)
HH grade II (n,%)	34 (69.4%)
HH grade III (n,%)	6 (4.9%)
HH grade IV (n,%)	4 (3.3%)

*ACA* anterior cerebral artery, *Acom* anterior communicating artery, *ICA* internal carotid artery, *Pcom* posterior communicating artery, *MCA* middle cerebral artery, *PCA* posterior cerebral artery, *PICA* posterior inferior cerebellar artery, *SAH* subarachnoid hemorrhage, *HH* Hunt-Hess.

The NFA stent was deployed in all patients in stent-assisted coiling of 129 aneurysms, with 134 stents deployed (Table 2). Immediately after endovascular embolization, the Raymond grade was I in 112 (86.8%) aneurysms, II in 8 (6.2%), and III in 9 (7.0%). No significant (P = 0.36) difference was detected in the Raymond occlusion degree between ruptured and unruptured aneurysms (Table 3).

**Table 2:** Treatment outcomes and complications.

Variables	Data
No. of stents deployed (n)	134
Raymond occlusion grades (n, %)	
Grade I	112 (86.8%)
Grade II	8 (6.2%)
Grade III	9 (7.0%)
Complications (n, %)	
Stent displacement	2 (1.6%)
Cerebral infarction	4 (3.3%)
Death	1 (0.8%)
Clinical follow-up (n, %)	
No. of patients	113 (92.6%)
Follow-up duration	6–30 (mean 21)
mRS grade 0	99 (87.6%)
mRS grade 1	7 (6.2%)
mRS grade 2	5 (4.4%)
mRS grade 3	2 (1.8%)
Angiographic follow-up (n, %)	
No. of patients	98 (80.3%)
Follow-up duration (m)	6–30 (mean 21)
No. of aneurysms	104 (80.6%)
Raymond grade I	94 (90.4%)
Raymond grade II	4 (3.8%)
Raymond grade III	6 (5.8%)
Occlusion outcome (n, %)	
Disappeared	93 (89.4%)
Unchanged	9 (8.7%)
Recurrent	2 (1.9%)

*mRS* modified Rankin Scale.

**Table 3:** Occlusion degrees of ruptured and unruptured aneurysms immediately after embolization.

Variables	Total aneurysms (n = 129)	Unruptured (n = 80)	Ruptured (n = 49)	P
No. of stents deployed (n)	134 (100%)	82 (61.2%)	52 (38.8%)	NA
Raymond occlusion grades (n, %)				
Grade I	112 (86.8%)	70 (87.5%)	42 (85.7%)	0.36
Grade II	8 (6.2%)	4 (5%)	4 (8.2%)	
Grade III	9 (7.0%)	6 (7.5%)	3 (6.1%)	

Periprocedural complications occurred in 7 (5.7%) patients, including stent displacement in 2 (1.6%) patients, thromboembolism and cerebral infarction in 4 (3.3%), and death in 1 (0.8%). In one patient with severe SAH and cerebral swelling, cerebral herniation occurred and caused death of the patient despite that the patient had been successfully embolized. Four patients had thrombosis which led to cerebral infarction post-embolization in spite of active thrombolysis. In three patients with thrombosis, navigation of the micro-guide wire and microcatheter into the target artery was very difficult, and long-time operation might result in formation of micro-thrombus to occlude distal arterial branches, causing subsequent cerebral infarction after embolization. In one patient, atherosclerotic plaques in cerebral arteries might be shed off during endovascular operation and caused distal arterial blockage and subsequent cerebral infarction. No significant (P = 0.42) difference was detected in the periprocedural complications between patients with unruptured and ruptured aneurysms (Table 4). Clinical follow-up was performed in 113 (92.6%) patients 6–30 (mean 21) months after embolization, with the mRS grade 0 in 99 (87.6%) patients, 1 in 7 (6.2%), 2 in 5 (4.4%), and 3 in 2 (1.8%). Good prognosis was in 111 (98.2%) patients while poor prognosis was in two (1.8%). No delayed hemorrhage or ischemic events took place. No significant (P = 0.45) difference was found in the clinical prognosis of mRS grades (Table 4).

**Table 4:** Periprocedural complications and clinical prognosis in patients with ruptured and unruptured aneurysms.

Variables	Total (n = 122)	Patients with unruptured aneurysms (n = 73)	Patients with ruptured aneurysms (n = 49)	P
Complications				
Stent displacement	2 (1.6%)	2 (2.7%)	0	0.42
Cerebral infarction	4 (3.3%)	3 (4.1%)	1 (2.0%)	
Death	1 (0.8%)	0	1 (2.0%)	
Clinical prognosis at follow-up				
No. of patients	113 (92.6%)	67 (91.8%)	46 (93.9%)	NA
Follow-up duration (m)	6–30 (mean 21)	6–28 (mean 19)	6–30 (mean 21)	NA
mRS grade 0	99 (87.6%)	61 (91.0%)	38 (82.6%)	0.45
mRS grade 1	7 (6.2%)	5 (7.5%)	2 (4.3%)	
mRS grade 2	5 (4.4%)	1 (1.5%)	4 (8.7%)	
mRS grade 3	2 (1.8%)	0	2 (4.3%)	

Digital subtraction angiography was conducted in 98 (80.3%) patients with 104 (80.6%) aneurysms 6–30 (mean 21) months after embolization. The Raymond grade was grade I in 94 (90.4%) aneurysms, II in 4 (3.8%), and III in 6 (5.8%). No significant (P = 0.14) difference existed in the Raymond grades at follow-up as compared with those immediately after embolization. Compared with the Raymond grades immediately after embolization, 93 (89.4%) aneurysms disappeared, 9 (8.7%) remained unchanged in the occlusion status, and 2 (1.9%) were recurrent. In one recurrent aneurysm which obtained Raymond grade II occlusion immediately after embolization but was recurrent at follow-up with the Raymond grade III, endovascular retreatment with coiling was performed and obtained complete occlusion. No severe in stent stenosis or occlusion was found at follow-up.

## Discussion

In this study investigating the effect and safety of the NFA stent in stent-assisted coiling of 129 wide-necked intracranial aneurysms, it was found that the NFA stent had a high aneurysm occlusion rate and a low complication rate in assisting coiling of wide-necked intracranial aneurysms even though further studies were necessary to prove this.

The NFA stent has some advantages, including a low profile, good trackability, easy passage through tortuous artery, and good conformability to vascular wall. Moreover, the stent has a very low metal coverage area of 6–12%, resulting in accurate localization and small shrinkage after deployment. For arteries with severe atherosclerosis, the stent can be directly delivered and deployed through a Gateway balloon catheter without a need of an exchange catheter, thus simplifying the procedure^9^.

In a multi-center study with 128 aneurysms in 128 patients^10^, 138 NFA stents were deployed in stent-assisted coiling of the aneurysms, resulting in a percentage of patients with Raymond grades I and II as high as 82.9% at discharge, an occlusion rate of aneurysms with Raymond grades I and II of 89.5% at the last follow-up, and a percentage of patients with mRS grades of 0–2 as high as 96.9% at the last follow-up^10^. In another study with 182 patients of anterior circulation aneurysms treated with the NFA stent-assisted coiling, 1-year angiographic follow-up of 153 patients showed a complete aneurysm occlusion rate (Raymond grade I) of 84.7%, no in stent stenosis > 50% nor retreatment even though eight (4.4%) patients died of severe ipsilateral stroke or neurological-related events^11^. For posterior circulation aneurysms with rich branching arteries, one study with 116 patients treated with the NFA stent-assisted coiling demonstrated a success stenting rate of 100%, a complete aneurysm occlusion rate at 1-year follow-up of 85.3% in 95 (81.9%) patients, a primary effectiveness outcome in 76.7%, an ipsilateral stroke rate of 4.3%, and a permanent neurological deficit rate of 1.7%^12^. These studies have revealed the safety and effectiveness in a short period of time. Similar outcomes were achieved in our study, which demonstrated Raymond grade I occlusion in 86.8% aneurysms and complications in 5.7% patients immediately after embolization. At follow-up of almost 2 years (21 months) after the embolization in our study, good prognosis with the mRS grades 0–2 was achieved in 98.2% patients, and Raymond grade I was present in 90.4% aneurysms.

Complications in endovascular embolization of intracranial aneurysms mainly include intraprocedural aneurysm rupture, thromboembolism, escape of coils, and stent displacement^10,12–14^. In the study by Burkhardt et al. using the NFA stent for the treatment of 128 patients with 128 wide-necked intracranial aneurysms^10^, technique-related complications occurred in six (4.7%) patients, including stenting failure in three patients, stent displacement in one, coil escape in one, and inability to coil the aneurysm after NFA deployment in one. In our study, four patients had thromboembolism. Besides difficult navigation of endovascular devices and long operation in three patients and presence of atherosclerotic plaques in the other patient, it may also probably be related to the following reasons. The blood in patients after subarachnoid hemorrhage was in a hypercoagulant state which has been shown to be one independent risk factor for intraprocedural thromboembolism in endovascular treatment of intracranial aneurysms^15^. Intimal injury or plaque detachment caused by endovascular operation in arteries with severe atherosclerosis may lead to distal embolism. Moreover, inadequate application or resistance of antiplatelet therapy during the periprocedural period may also cause thrombosis and distal embolism. In a study comparing the technical success and complication rates of the NFA (n = 37) and the Low-profile Visualized Intraluminal Support Junior (LVIS Jr) (n = 27) stents by Gross et al.^16^, the rate of symptomatic postoperative complication rate was not significantly (P = 0.15) different in the NFA (3%) versus the LVIS Jr (15%) stent, the aneurysm occlusion rate of the Raymond grade I or II at follow-up was significantly (P = 0.04) better in the NFA (100%) versus the LVIS Jr (81%) stent, and the in stent stenosis rate at follow-up angiography was significantly (P = 0.04) fewer in the NFA stent compared with that in the LVIS Jr stent (19%). These low complication and in stent stenosis rates were probably associated with the low metal coverage area and good conformability of the NFA stent. In our study, the complication rate was also low (5.7%), including stent displacement in two (1.6%) patients, thromboembolism in 4 (3.3%), and death in one (0.8%).

Other stents have also been used in endovascular treatment of wide-necked cerebral aneurysms. The second-generation Enterprise 2 stent (Codman Neurovascular, Miami Lakes, FL, USA) features improved maximal diameter (5.0 mm) and stent strut geometry to reduce the incidence of operative complications by achieving better stent-vessel wall apposition^17,18^. In a study investigating the effects of the Enterprise 2 stent in 106 wide-necked aneurysms in 106 patients by Zhou et al.^17^, the stent was successfully deployed in all patients, perioperative complications occurred in 10 patients (9.4%) (including cerebral infarction in 6), follow-up angiographic aneurysm occlusion was satisfactory (Raymond grades I and II) in 89 (93.7%), and poor clinical outcome (mRS ≥ 3) at the last follow-up was seen in seven patients. In a study exploring the effect of the Leo Baby stent (Balt Extrusion, Montmorency, France) for assisted coiling in 131 patients with 135 aneurysms^19^, the technical success was achieved in all cases (100%), complete occlusion was obtained in 111 aneurysms (82.2%), neck remnants in 19 (14.1%), and residual sac in 5 (3.7%) immediately after embolization. Procedure-related complications occurred in 14 cases (10.3%), including 13 (9.6%) thromboembolic complications and 1 (0.7%) hemorrhagic complication. In 6-month follow-up angiography, complete occlusion was achieved in 102 (96.2%) aneurysms, the favorable clinical outcomes (mRS 0–2) reached 91.6%, and the mortality rate was 4.6%. The outcomes of these stents were similar to those of the NFA in our study. Nonetheless, large cohorts of patients are needed to confirm these outcomes.

Flow diverters have been approved for endovascular treatment of medium-sized cerebral aneurysms, and in the current literature, flow diverters have been applied to treat both small- and medium-sized cerebral aneurysms^20–23^. In a study investigating the Pipeline flow diverter alone or combined with coiling for treatment of cerebral aneurysms with different sizes in a large cohort of 1171 patients with 1322 aneurysms^23^, angiographic follow-up did not show any difference in the aneurysm occlusion rate between the Pipeline Embolizing Device (PED) alone and PED combined with coiling in small aneurysm ≤ 7 mm in diameter (79.1% vs 88.4%, respectively) even though a significant increase in the ischemic complication rate was detected in the treatment with the PED combined with coiling (19.3% vs. 8.3%, P = 0.0001). For medium and large or giant saccular aneurysms, the PED combined with coiling had significantly increased the aneurysm occlusion rate (88.8% vs. 74.7% with a P < 0.0001 for medium sized aneurysms ≤ 15 mm and 86.9% vs. 72.9% with a P = 0.018 for large and giant aneurysms > 15 mm) as compared with the PED deployment alone even though the complication rate was not significantly different. In our study, we did not use flow diverters, and only the NFA stent was used. When using the conventional intracranial stents like the NFA stent for endovascular treatment of cerebral aneurysms, small- and medium-sized aneurysms are the appropriate indications of treatment. Nonetheless, in stent-assisted coiling of large and giant aneurysms > 15 mm in diameter, dense packing with coils of the large aneurysm cavity may lead to mass effect and subsequent clinical symptoms. In these cases with large aneurysms, flow diverters may be better because these diverters alone without combined coiling can divert blood flow from direct impinging the aneurysm neck and dome, thus eliminating the possible mass effect and subsequent clinical symptoms caused by coil dense packing.

In our study, dual antiplatelet therapy was conducted for patients with unruptured cerebral aneurysms composing aspirin 100 mg/day and clopidogrel 75 mg/day for 5–7 days before endovascular embolization. After the embolization procedure, the dual antiplatelet therapy was continued for 3 months before switching to aspirin alone. This dual antiplatelet therapy is common for unruptured aneurysms^24–27^ and has become a standard regimen to prevent thromboembolic events in stent-assisted endovascular treatment of cerebral aneurysms^27–30^. For patients with ruptured cerebral aneurysms, tirofiban was used intraprocedurally when deploying a stent, and later, dual antiplatelet therapy with clopidogrel and aspirin was administered as in patients with unruptured cerebral aneurysms in our study. This antiplatelet medication is also used to prevent thromboembolic events in stent-assisted coiling of ruptured cerebral aneurysms and is commonly used in stent-assisted endovascular embolization of acutely-ruptured cerebral aneurysms^31–35^. Tirofiban acts very rapidly and is able to prevent thromboembolic events in endovascular treatment of ruptured cerebral aneurysms. After the ruptured aneurysms were embolized with stent-assisted coiling, routine dual antiplatelet therapy with aspirin and clopidogrel can be conducted to prevent possible thromboembolic events later.

Some limitations existed in this study, including the retrospective and one-center study design, no control or randomization, Chinese patients enrolled, a small sample, and short follow-up period, which may all affect the generalization of this study. Future prospective, randomized, controlled clinical trials with inclusion of multiple medical centers and multiple races and ethnicities will have to be performed to obtain better outcomes.

In conclusion, the NFA stent may result in a high aneurysm occlusion rate and a low complication rate in assisting coiling of wide-necked intracranial aneurysms, and the NFA stent has provided a new therapeutic option for endovascular treatment of wide-necked intracranial aneurysms even though further studies are necessary to prove this.

## Data Availability

Data are available from the corresponding author on reasonable request.
